# Optimized Electrode Placements for Non-invasive Electrical Stimulation of the Olfactory Bulb and Olfactory Mucosa

**DOI:** 10.3389/fnins.2020.581503

**Published:** 2020-11-12

**Authors:** Yusuf Ozgur Cakmak, Kamran Nazim, Chris Thomas, Abhishek Datta

**Affiliations:** ^1^Department of Anatomy, University of Otago, Dunedin, New Zealand; ^2^Brain Health Research Center, Dunedin, New Zealand; ^3^Medical Technologies Center of Research Excellence, Auckland, New Zealand; ^4^Centre for Health Systems and Technology, Dunedin, New Zealand; ^5^Research and Development, Soterix Medical, New York, NY, United States; ^6^City College of New York, New York, NY, United States

**Keywords:** olfactory perception, olfaction, non-invasive electrical stimulation, smell, odor, wearable, Parkinson’s disease, Alzheimer’s disease

## Abstract

The olfactory system is known to be dysfunctional in the early stages of Parkinson’s disease (PD) and Alzheimer’s disease (AD). It is also shown that intact olfactory function can be a key role player for regaining consciousness after brain injuries. Modulation of the olfactory regions has been attempted successfully with electrical stimulation over the years, either directly (transethmoidally, intraoperatively, internasally, etc.) or indirectly through the vagus nerve. We sought to develop a means of delivering optimized electrical stimulation to the olfactory region in a non-invasive fashion and in a way that is simpler, easier, and less cumbersome. The ultimate goal was to develop a system that would allow easier testing in future clinical trials presenting an opportunity to fully develop this potential treatment option. We devised six potential electrode placements leveraging commonly accepted facts of electrical stimulation, easier access through relatively higher conductive pathways into the brain, and practicality. Using an ultra-high-resolution finite element model, we screened each one of these montages for their ability to target the olfactory regions primarily and thereafter for select sub-cortical regions implicated in the pathogenesis of PD and AD. Modeling results indicate that some placements do result in inducing meaningful electric field magnitudes in the regions of interest. A practical headgear concept is proposed to realize the most ideal configuration. Our results pave the way for developing the first non-invasive electrical stimulation wearable system for targeting the olfactory regions which can help to alleviate the symptoms or suppress the progression of these neurological disorders.

## Introduction

The olfactory system, which is one of the cardinal sensory gateways to the brain, is known to be dysfunctional in the very early stages of Parkinson’s disease (PD) and Alzheimer’s disease (AD) (Rezek, 1987; Mesholam et al., 1998; Duff et al., 2002; Motomura and Tomota, 2006; Fusetti et al., 2010; Doty, 2012; Alves et al., 2014; Doty and Kamath, 2014). The pathological Lewy bodies are also shown in the olfactory bulb (OB) (Li et al., 2016) and distributed to motor centers with the progression of the disease in PD (Braak et al., 2003; Ross et al., 2008; Doty, 2012; Li et al., 2016). In addition, pathological tau and amyloid protein accumulations have been demonstrated in the olfactory epithelium/mucosa (OE) (Lee et al., 1993) and olfactory end-terminals including the entorhinal cortex (EC) in AD patients (Näslund et al., 2000; Desikan et al., 2012; Khan et al., 2014). In a recent research, it has also been shown that 100% (specificity) of patients who respond to olfactory stimulus in the unresponsive states (or low level of consciousness) regained consciousness (Arzi et al., 2020).

In the context of the underlined dysfunctions of the olfactory system in the early stages of AD, PD, and also for vegetative states, the stimulation of the olfactory nerve and system has gained significance as a potential neuromodulation target. There have been attempts to deliver electrical stimulation *directly* to the OE via positioning the stimulation electrodes as close as possible to the “target.” These placements all involved electrode insertion through the nostril, but studies employed a range of stimulation dose, application precision, and desired olfactory targets. Straschill et al. (1983) performed stimulation by an electrode attached to a rhinoscope and delivered 2 mA/0.5 ms pulses. Ishimaru et al. (1997, 2002), in several studies from 1997 to the most recent study in 2002, used a bipolar stimulating electrode (with no indication of guiding mechanism used) and also delivered 2 mA/0.5 ms pulses. Weiss et al. (2016) and more recently Holbrook et al. (2019) both used an electrode placed with endoscopic guidance. Weiss tested a range of stimulation parameters applied at currents ranging between 50 and 800 μA targeting the ventral surface of the middle turbinate. Holbrook et al. (2019) used constant square wave pulses with gradually increasing intensity from 1 to 20 mA and importantly targeted the OB through the thin bone of the cribriform plate. It is important to note that Holbrook and colleagues were able to access the thin bone as they only included a patient group with previous complete ethmoidectomies. None of the prior work to the Holbrook study were able to evoke any perception of smell in spite of generation of olfactory-evoked potentials (potential change on the scalp) and fMRI-determined activity in the primary olfactory cortex (Straschill et al., 1983; Ishimaru et al., 1997, 2002; Weiss et al., 2016). However, the direct stimulation of the OB and utilization of stimulation parameters based on neurology and neurosurgical studies likely explained the first demonstration of smell sensation in the Holbrook study. In addition, there have been efforts to affect olfactory function by using subdural electrodes (targeting frontal lobes proximal to the OB) (Kumar et al., 2012), and a review of stimulations performed using depth electrodes indicated olfactory sensations upon direct stimulation of the mid-dorsal insula (Mazzola et al., 2017).

Peripheral nerve stimulation has also been trialed to modulate olfactory function. García-Díaz et al. (1984) reported modulation with 80 Hz invasive electrical stimulation of the vagus nerve in animals. Our group (Maharjan et al., 2018) reported the modulation of olfactory function with 80-Hz non-invasive electrical stimulation of the auricular vagus nerve in humans for the first time in literature. We also reported increased perfusion in the right (contralateral) orbitofrontal cortex (OFC) regions only for high-frequency stimulation. In a follow-up study, we also demonstrated that median nerve stimulation is capable of suppressing the olfactory function in humans (Maharjan et al., 2019).

Subsequent to all the aforementioned work, the next logical question was to explore not only direct targeting of the olfactory regions but also in a fashion that is simpler, user friendly, safe, comfortable, and less cumbersome than a nasal insertion or an epidural approach and that could therefore be tested on a larger patient group. The ability to run larger trials would allow investigators an opportunity to demonstrate clinically meaningful effect sizes, establish mechanism of action, and determine long-term effects.

We therefore considered non-invasive scalp electrode placements that are strategically proximal to the intended target (i.e., nose regions and forehead) and evaluated them for their targeting capability. The ultimate goal was to determine the most ideal placement that would allow developing the *first* future wearable system for olfactory neuromodulation. The placements could also involve distal electrodes as long as the dominant current flow (between the stimulation electrodes due to laws of physics) would *traverse* the olfactory regions. We started by evaluating six novel candidate electrode placements based on some commonly accepted facts of electrical stimulation. First being that placing electrodes proximal to each other enforces restricted current flow pattern thereby increasing focality but at the expense of increased total injected current (Datta et al., 2008). The opposite being the case for electrodes placed distally. Second is the ability to “fashion” current flow direction by simple placement choice. For instance, electrodes placed on left and right temporal locations will result in dominant flow in the left–right and vice versa direction depending on monophasic/biphasic stimulation waveform. The last but not the least, we also consider ease of administration—for instance, montage avoiding hair regions to overcome potential limitations of electrodes on a hairy zone in a wearable device is more attractive.

Our overall goal in this study was to simulate the current flow distribution in the olfactory system, including the OE, OB, and EC and also in the entire brain, and thereby determine the most promising placement for a future clinical study. Additionally, our results would help serve two purposes—(a) determine whether exemplary weak scalp current (1 mA) considered here can even reach the olfactory regions to influence neural activity even though applied current can be scaled and (b) if current of sufficient magnitude does reach the regions of interest, how do we proceed in developing a wearable device for PD and AD patients to be used in their daily routine? We further note that our aim was to determine optimized electrode placements valid for the range of aforementioned intensities and waveforms (pulses and sine) used for stimulation of the olfactory regions thus far.

We developed an ultra-high-resolution model based on a 0.5 mm isotropic resolution dataset which was necessary to resolve the tiny structures of interest in this study, i.e., the OB and the OE. We determined induced surface and cross-sectional electric field (EF) maps for the entire brain for each of the electrode placements considered. We also compared focality relative to an EF threshold value to enable a quantitative comparison. Furthermore, current flows in the OB, OE, basal ganglia, and hippocampus were individually analyzed and additional metrics of polarization considered for a subset of the configurations.

## Materials and Methods

### Data Considered and Pre-processing

The ultra-high-resolution head and neck model (MIDA: multimodal imaging-based detailed anatomical) available through the IT’IS Foundation was used in this study (Iacono et al., 2015). The MIDA model was merged with a cropped version of Duke (Visible Human Project) to extend the model until the level of the chest. In order to do so, the Duke model was first cropped at the level of the chest and then imported into the same anatomical space as the MIDA model. The Duke model geometry was then aligned with the neck region of the MIDA model and the contact interfaces modified by applying appropriate filters to minimize any abrupt transition between the two models. This process ensured a simplified geometry at the merged sections leading to a simpler mesh.

### Tissue Segmentation and Electrode Placements

The nifti (.nii) color masks from the MIDA model were first processed in MATLAB to re-create segmentation masks based on intensity values. These masks were then imported into Simpleware (Synopsys Ltd., CA, United States), and any errors in continuity and anatomical details were manually corrected for Bikson and Datta (2012), Datta et al. (2012), Haberbosch et al. (2019). Masks with similar electrical conductivities were then merged to a single compartment excluding the regions of interest (OB and OE) in order to perform detailed individual current flow analysis through them.

The OE was extracted from the upper third of the inner mucosa region of the MIDA model using the 3D editing tool in Simpleware. The area was determined by using a combination of visual references as a guide (Purves et al., 2001; Sawa, 2015).

The stimulation electrodes and the conductive media (gel) were created as computer-aided design (CAD) models (STL files) having either circular or oval disk shapes and were positioned interactively within the image dataset. The circular disks were smaller (6-mm diameter) while the oval disks bigger (long axis = 25 mm; short axis = 20 mm). All electrode disks had a thickness of approximately 1 mm and were modeled as conductors with the conductivity of copper. The thickness of the gel disks were approximately 1 mm and were assumed to have the conductivity of a typical conductive gel used for electrical stimulation applications.

The following six novel montages were simulated:

1. Nose bridge + upper posterior (Montage 1): Two circular disks were placed on the immediate right and left of the nose bridge and two oval-shaped disks placed at the back of the head. The back electrodes were positioned corresponding to 1 cm to the left and right of the midline at the level of POz (location corresponding to the 10–10 EEG layout).
2. Upper forehead + lower posterior (Montage 2): Two circular disks were positioned on the forehead with two circular disc electrodes positioned on the lower back of the head. The forehead electrodes were placed about 3 cm above the nasion and about 1.5 cm away from each other or 0.75 cm on either side of the midline right below Fpz. The lower posterior electrodes were placed about 1 cm left and right of the midline at the level of the inion.
3. Lower forehead + neck posterior (Montage 3): Three circular disks were positioned on the lower forehead (slightly above the eyebrow) with four oval-shaped disk electrodes positioned on the upper neck. The disks were placed 1.5 cm apart from each other, about 1 cm above the nasion, with the central electrode along the midline. The four oval-shaped electrodes were positioned about 4.5 cm below the level of Iz and spaced about 4 cm from each other. The posterior electrodes were also positioned symmetrically from the midline.
4. Lower forehead and nose bridge + neck posterior (Montage 4): Three circular disks positioned similarly on the lower forehead as Montage 3 including nose bridge electrodes similar to Montage 1. This combination was paired with two oval-shaped electrodes positioned at similar location to Montage 3.
5. Lower forehead + behind ear (Montage 5): Three circular disks positioned similarly on the lower forehead as Montage 3 configuration and two electrodes behind the ear avoiding the hairline. The behind ear electrodes corresponded to P9 and P10 of the 10–10 EEG layout.
6. Lower forehead and nose bridge + neck posterior (Montage 6): Same as Montage 4 but with one electrode on the lower forehead positioned along midline. This combination was paired with two oval-shaped electrodes positioned at a similar location to Montage 4.

The integrated CAD files were converted to masks, and appropriate filters and Boolean operations applied to ensure no overlapping tissue masks.

### Computer Model Development and Computation

Adaptive meshes derived from the segmentation and the CAD masks are then created for finite element (FE) analysis in COMSOL Multiphysics (Burlington, MA, United States). The final models on an average comprised >30 million elements with >50 million degrees of freedom.

The studies targeting the olfactory system have typically considered *T* = 0.5 ms duration pulses. A modification of the standard Laplace’s equation incorporating a reactive component to account for the frequency (spectral content) is appropriate to determine the induced EF:

$$\begin{array}{c} \nabla \cdot \left(\sigma +j\omega \varepsilon \right)\nabla V=0 \end{array}$$

where ε is permittivity and ω is angular frequency.

The corresponding Fourier magnitude spectrum of a 0.5-ms duration pulse indicates power concentrated from 0 to 2 kHz with 2 kHz reflecting the first zero crossing (1/*T*). The consideration of tissue properties (conductivity and permittivity) at 1 kHz (half of the first zero crossing) reveals that the real component of equation dominates such that the reactive component can be ignored (Gabriel et al., 1996; Edwards et al., 2013). This results in a simplified standard Laplace’s equation:

$$\begin{array}{c} \nabla \cdot \left(\sigma \nabla V\right)=0 \end{array}$$

that considers purely conductive properties. Furthermore, tissue conductivity properties at 1 kHz are not substantially different than 0 Hz and have been shown experimentally to not result in any scalp potential differences (Datta et al., 2013). Taken together, 0 Hz or DC conductivity values are therefore considered here. Table 1 lists the representative isotropic average electrical conductivities assigned to the different tissue compartments and the electrode materials (in S/m). The boundary conditions used were as follows: (1) inward current flow = Jn (normal current density) applied to the exposed surface of all the anterior electrodes considered in the individual montages (nose bridge, upper forehead, and lower forehead), (2) ground applied to the exposed surface of all posterior electrodes considered in the individual montages (upper posterior, lower posterior, neck posterior, and behind ear), and (3) all other external surfaces treated as insulated. The current density corresponding to 1 mA exemplary total injected current was considered for all montages.

**TABLE 1:** Assigned electrical conductivities.

Tissue compartment/electrode material	Electrical conductivity (S/m)
Scalp	0.465
Muscle	0.35
Skull	0.01
CSF	1.65
Gray matter	0.276
White matter	0.126
Fat	0.04
Blood	0.7
Eye	1.65
Air	1e-7
Conductive gel	0.3
Electrode (material)	5.8e7
Cartilage	1.01
Intestines	0.164
Mucosa	0.0004
Olfactory bulb	0.126
Olfactory epithelium and olfactory mucosa	0.0004

### Data Analysis

Electric field (EF) magnitude plots on the cortical surface and on OE were generated for each of the six novel montages. To facilitate a quantitative comparison, we determined stimulation focality by percentage volume of OE subject to EF magnitude greater than EF threshold value (arbitrarily chosen range but expected for 1 mA intensity considered). For each of the selected optimal montages, surface EF plots were generated for the OB and the OE. In addition, cross-sectional EF plots were generated to visualize depth modulation. Finally, in addition to EF, we considered two other drivers of neuronal polarization along an exemplary axon in the OE (EF in the axon direction and the activating function) to further elucidate differences between the configurations with respect to these “driving functions” (Warman et al., 1992; Rubinstein, 1993; McIntyre and Grill, 1999). This exemplary axon orientation was simulated to mimic actual anatomical orientation. We note that since the OB primarily consists of neuron bodies, we do not consider any other driver of neuronal polarization in the OB other than the electric field (Datta et al., 2008).

## Results

**FIGURE 1:**
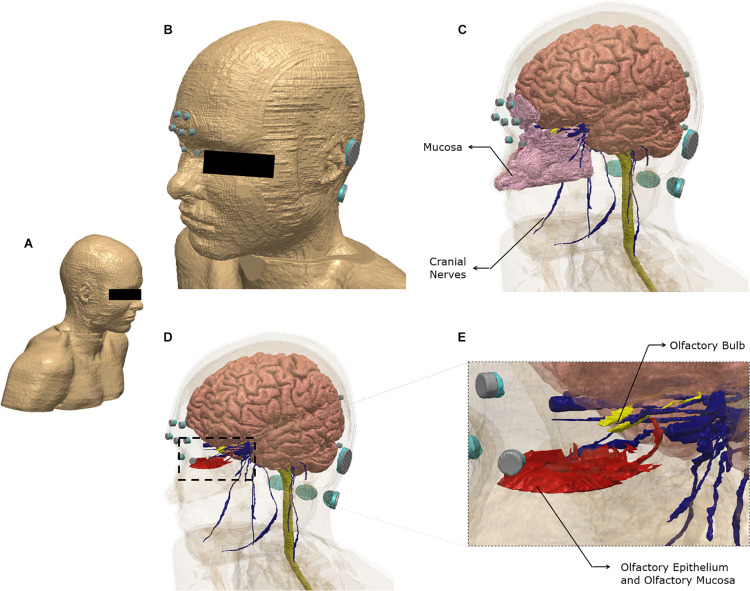
Model geometry and segmentation masks. The ultra-high-resolution multimodal imaging-based detailed anatomical (MIDA) model was fused with neck and shoulder sections from a model derived from the Visible Human Project. See section “Materials and Methods” for additional information **(A)** The full extent of the geometry considered. **(B)** Scalp mask with the electrodes used for the finite element model. Each electrode (silver) is interfaced with tissue via conductive gel (light blue). Note some electrodes/gel are not visible because of the view chosen and the need to obscure the eye region. **(C)** Additional masks shown include gray matter, white matter including the brain stem and spinal cord, mucosa, and cranial nerves. **(D)** The masks in the nose region are made semi-transparent to show the olfactory epithelium and the olfactory bulb. The olfactory epithelium mask includes the olfactory mucosa mask. **(E)** The dashed section in **(D)** is expanded to highlight segmentation detail. Note that items **(B–E)** were plotted to the same perspective.

Brain current flow (electric field) was predicted using an FE model derived from an ultra-high-resolution dataset (see section “Materials and Methods”). Figure 1 illustrates the FE model geometry considered in the study and select 3-dimensional segmented tissue masks.

**FIGURE 2:**
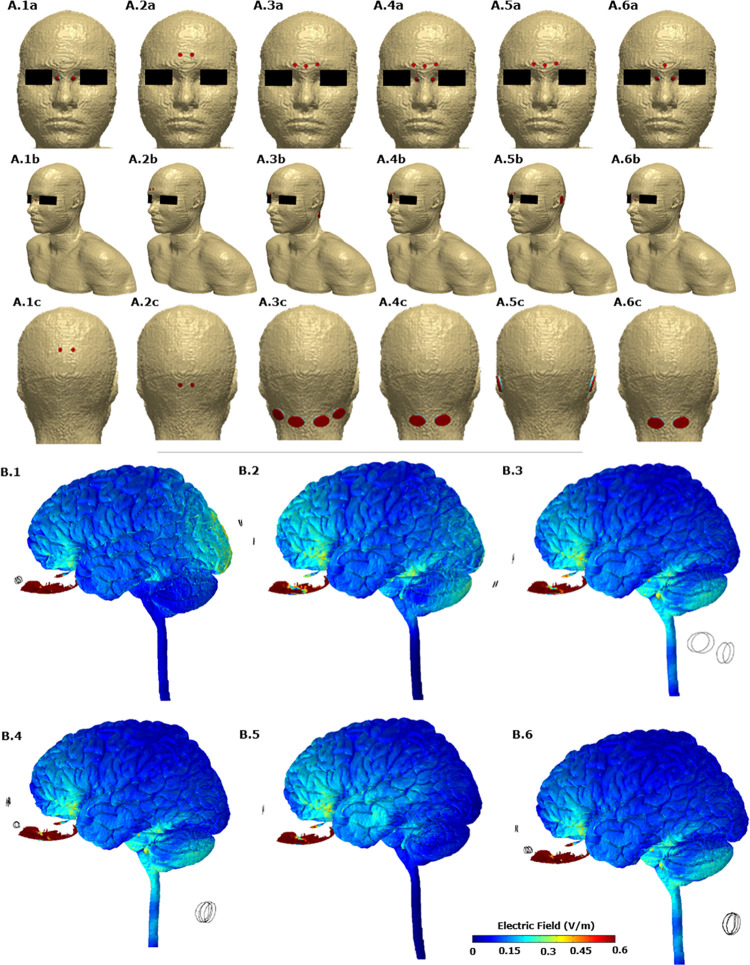
**(A)** The six candidate electrode configurations evaluated in the study. A.1, Montage 1: nose bridge + upper posterior; A.2, Montage 2: upper forehead + lower posterior; A.3, Montage 3: lower forehead + neck posterior; A.4, Montage 4: lower forehead and nose bridge + neck posterior; A.5, montage 5: lower forehead + behind ear; A.6, Montage 6: lower forehead and nose bridge + neck posterior. The actual FE model that was computed is shown in the middle row (A.1b, A.2b, A.3b, A.4b, A.5b, and A.6b). **(B)** Electric field plots on the cortical surface and on the olfactory epithelium for each of the six initial *candidate* electrode configurations. B.1 plot corresponds to A.1. Similarly B.2 plot corresponds to A.2 and so forth. Only the left lateral view is shown.

Figures 2A.1–A.6 allows us to visualize the exact electrode placements considered with respect to the anatomy along with electrode dimension and shape. For each one of the six initial candidate montages, we first calculated the induced electric field (EF) magnitude on the brain surface (Figures 2B.1–B.6). These plots allow a direct comparison of the relative surface focality and thereby screening for montages with higher focality for additional analysis. We observed that Montage 1 (A1 in Figure 2), Montage 4 (A4 in Figure 2), and Montage 6 (A6 in Figure 2) result in increased current flow in the OE regions. Each one of these montages comprise of electrodes positioned on either side of the nose bridge indicating that the proximity to the target ROI plays a predominant role in increased focality.

**FIGURE 3:**
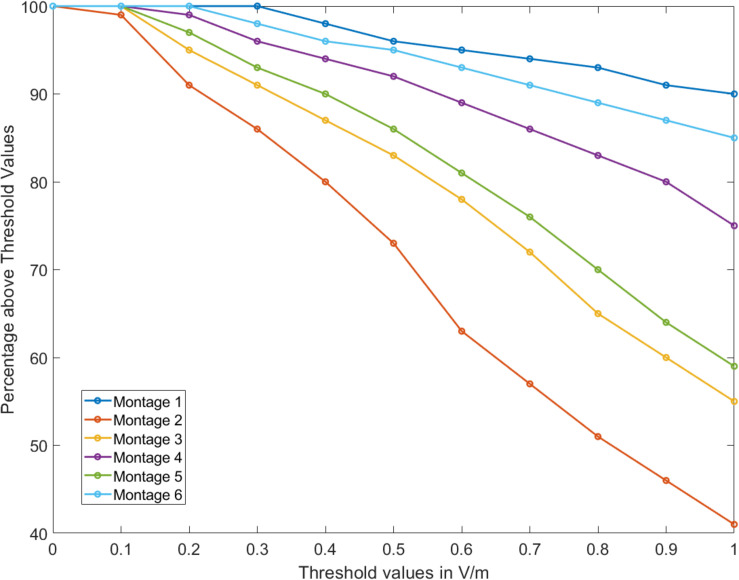
Percentage volume above electric field threshold. For all montages considered, percentage of the olfactory epithelium (OE) volume above a given threshold of electric field was calculated from 0 to 1 V/m.

Further, the OE volume percentage plots exceeding a particular EF threshold indicate that for Montages 1, 4, and 6, greater than 75% of the volume is subject to a value >1 V/m (Figure 3). Montage 2 resulted in the lowest volume percentage > 1 V/m (∼40%) confirming the observation from the surface plot (Figure 2B.2) showing reduced EF in the OE regions in comparison to the other montages. The underlying motivation in considering montages involving the forehead (Montages 2, 3, and 5 represented as A2, A3, and A5, respectively, in Figure 2) was the potential to “force” current flow in a downward trajectory toward the lower return electrodes and thereby targeting the ROI in its path. While there is some current flow in the ROI, the montages comprising the nose bridge electrode clearly hold more promise and were subjected to further analysis. This extended evaluation allowed us to perform a detailed current flow analysis through not only the OE but also other relevant structures of interest (OB, basal ganglia, and the hippocampus).

**FIGURE 4:**
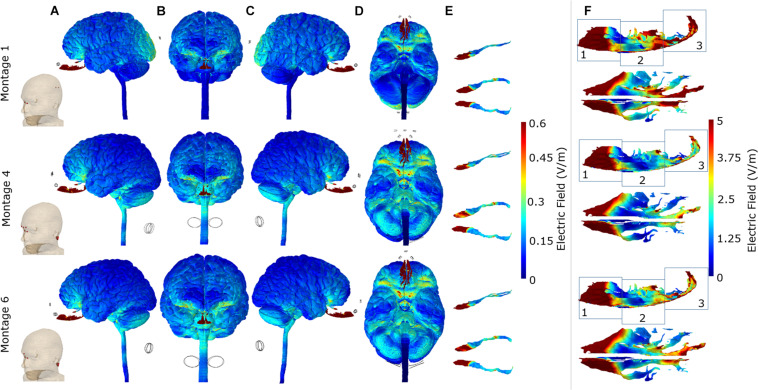
Detailed current flow analysis of the three *optimal* montages. First row: Montage 1, second row: Montage 4, and third row: Montage 6. **(A)** Left lateral view; **(B)** front view; **(C)** right lateral view; **(D)** bottom view; **(E)** side and top view of the olfactory bulb; **(F)** side and top view of the olfactory epithelium. Note that the current flow maps for the olfactory epithelium are plotted to a reduced maximum value to better highlight differences amongst the three optimal montages. In addition, we partition the side view plots of the OE into three different sub-sections (1, 2, and 3) to facilitate easier comparison across the montages.

For each of the three optimal montages, we considered surface EF plots for the whole brain and individually for the OB and OE (Figure 4). The whole brain plots confirmed the expected dominant downward current flow for Montages 4 and 6 given the location of the electrodes. This is shown by the increased current flow through the orbitofrontal cortex and ending at the lower surface of the cerebellum in these two montages. However, Montage 1 showed increased current flow through the orbitofrontal cortex but ending at the occipital lobe (visual cortex). With respect to targeting the OB, the electrical current flow modeling virtually demonstrated the potential efficacy of all the three montages to direct the electrical current into the OB. In the context of the OE, all of the three montages are also capable of effectively directing current on the anterior 1/3 sub-section of the OE (Figure 4F). On the other hand, we observed that Montage 1 led to the most widespread current flow in the 1/3 middle and 1/3 posterior sub-sections (regions 2 and 3) of the OE. Montage 6 had moderate effect on the 1/3 posterior sub-section of the OE, whereas the Montage 4 demonstrated the least influence on the middle and posterior OE among the three montages (Figure 4F).

**FIGURE 5:**
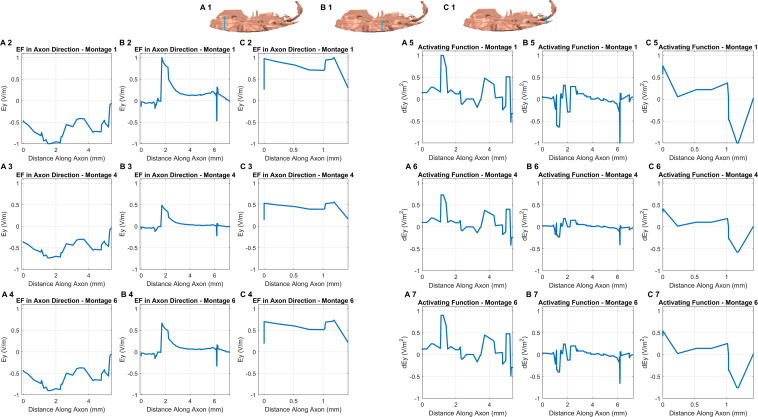
EF along the axon and activating function. We first assumed an exemplary axon in each of the three sub-sections (blue line in each section shows the location) and in an orientation reflective of the actual anatomical situation (A1, B1,C1). The axon considered in the third sub-compartment was the shortest given the lack of tissue in the region. For each of the axons, we considered the EF component aligned with the axon for each montage (section on the left). We repeated the same by considering the activating function (section on the right). The plots were normalized relative to the highest induced value (observed in Montage 1) to facilitate easier comparison.

The consideration of additional potential drivers of polarization along an exemplary axon in each of the three sub-sections in the OE helps in further studying the differences among the optimal montages (Figure 5). Further, all plots were normalized relative to the highest induced value (observed in Montage 1) to enable easier comparison. While the EF component aligned with the axon plots reveals different profiles in each sub-section (see any row), we observed similar profiles across each of the three optimal montages (see any one column). We also noted a similar pattern when considering the derivative of EF along the axon or the activating function. Each montage indicated a similar profile but the profile was different in different sub-sections. This is likely explained due to the similar electrode configuration employed with all, involving a combination of electrodes positioned at the front (around the nose bridge) with electrodes at the back of the head, resulting in similar overall current flow pattern. This results in a similar voltage profile in any one sub-compartment which naturally manifests into similar polarization metrics as both EF component along the axon and the activating function are related to voltage. The notable difference across montages is the highest induced value for Montage 1 for both EF in the axon direction and the normalized activating function. This makes Montage 1 the ideal candidate when considering the highest likelihood for activation of the OE across all montages considered.

**FIGURE 6:**
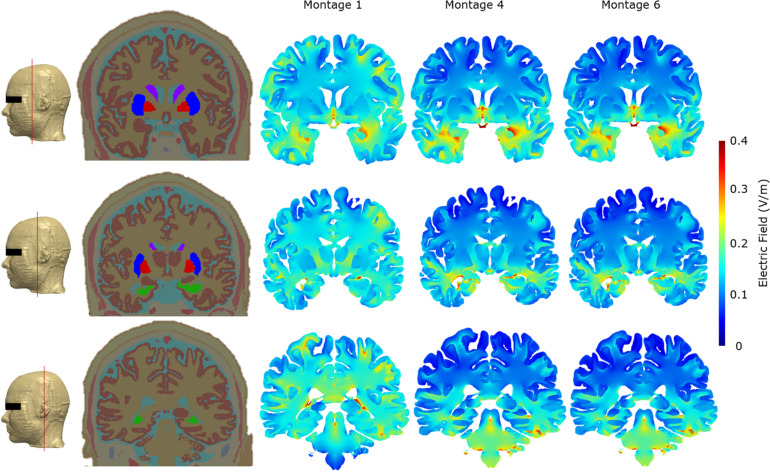
Cross-section segmentation and brain electric field plots for the three *optimal* montages. All false color maps were generated between 0 and 0.4 V/m. The slices were chosen to evaluate cross-sectional current flow in the basal ganglia and hippocampus regions. The first column shows the level at which the representative coronal images were taken in the FE model. The second column shows the coronal whole head tissue segmentation images; purple, caudate nucleus; blue, putamen; red, globus pallidus; green, hippocampus; pink, substantia nigra. The third column shows Montage 1, the fourth column shows Montage 4, and the fifth column shows Montage 6.

As part of the final detailed analysis, we considered representative coronal 2D slices at three different sections to highlight current flow patterns through the basal ganglia (considering their role in Parkinson’s disease) and particularly the hippocampus/parahippocampus/EC as the secondary terminals of olfactory pathways and considering their reported roles in the pathogenesis of the early stages of Alzheimer’s disease. Our simulations indicate that Montage 1 induced higher EF magnitude overall in the brain—particularly on the cortical structures including motor and somatosensory cortices (Figure 6). On the other hand, Montages 4 and 6 have the least impact over upper segments of the cerebrum and cortical structures including the motor and somatosensory cortices but have more localized and prominent effect over the hippocampal/parahippocampal/EC. For the structures comprising the basal ganglia and internal capsule, Montage 1 was found to have higher induced current flow in in comparison to Montages 4 and 6 (Figure 6).

In brief, all three montages were capable of stimulation of the OB, 1/3 anterior sub-segment of OE, and orbitofrontal cortex, but due to the dominant downward trajectory of current flow for Montages 4 and 6, higher EF was induced in the hippocampal and parahippocampal/EC cortex and terminated at the cerebellum. However, Montage 1 directed the current to the overall cerebrum with relatively less impact on the hippocampal/parahippocampus/EC and more impact on the motor/sensory cortex (in comparison to Montages 4 and 6) and the current terminating at the occipital cortex but not in the cerebellum (Figures 4, 6).

**FIGURE 7:**
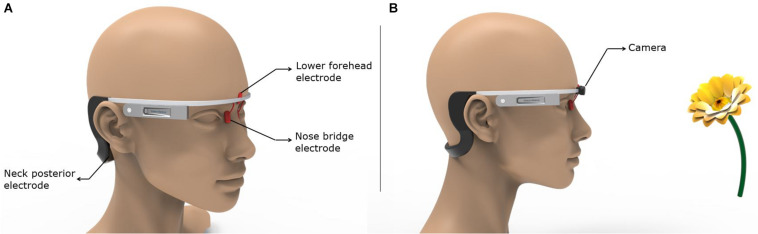
Implementation of the most easily administered and optimal montage. The key to the successful use of the easiest to use and optimal montage (Montage 6) is ensuring holding the nose bridge electrodes at their intended location in a robust fashion. Other factors such as comfort, unobtrusiveness, weight, etc. will play an equally important role. **(A)** We borrow the Google Glass design to propose a means on which an initial prototype could be based. **(B)** The same concept as shown in **(A)** is combined with a camera that could be used to leverage object recognition.

Finally, we propose a means to realize the most easily administered configuration in a future clinical trial targeting the olfactory regions. Montage 6 is considered the most practical and easy-to-use option given the fully hair-free electrode montage configuration and lower number of electrodes. The lower forehead and the nose bridge electrodes could be held using a headgear similar to the Google Glass concept (Figure 7A). The neck posterior electrodes could either be attached to the end of the arms wrapping around the ears or realized via using commonly available hydrogel electrodes in standalone fashion. Figure 7B demonstrates a concept idea for potential future applications with embedded camera and machine learning algorithms for distance recognition to an object (e.g., a flower) with an odor and defining the object with machine learning to induce different odor sensations with specified stimulation parameters.

## Discussion

Olfactory dysfunction is known to be one of the earliest symptoms of AD, and it has been shown that from OE to EC, all the olfactory network structures demonstrate pathology in AD; neurofibrillary tangles are observed in the OB at the early stages of AD (Braak and Braak, 1991; Price et al., 1991; Van Hoesen et al., 1991; Saiz-Sánchez et al., 2011; Kovács et al., 2001) as well as the tau pathologies in the OE (Lee et al., 1993). EC is involved in memory and cognitive functions, and it is one of the first zones that has shown pathological amyloid protein accumulations and neurofibrillary tangles as well as cell loss in AD (Stranahan and Mattson, 2010; Criscuolo et al., 2017). EC has two sub-divisions: the medial entorhinal cortex (MEC) and lateral entorhinal cortex (LEC). While the MEC is known to be spatially modulated (Fyhn et al., 2004; Hafting et al., 2005; Hargreaves et al., 2005; Sargolini et al., 2006; Criscuolo et al., 2017), the LEC cells also respond to olfactory stimulus (Habets et al., 1980a,b; Eichenbaum et al., 2007; Criscuolo et al., 2017). In other words, only the LEC responds to olfactory stimuli and it is the gateway to the hippocampus that drives memory (Gómez-Isla et al., 1996). It has been reported that EC is primarily affected in preclinical AD (Khan et al., 2014) and is tightly related to memory deficiencies in AD (Nagy et al., 1996). The above-mentioned pathologies in olfactory structures and its terminals including LEC emphasize these structures as the potential targets of non-invasive neurostimulation in the early stages of AD.

To our knowledge, non-invasive transcutaneous/transcranial electrical stimulation (tES) of the OB and OE with a wearable system has not been investigated yet. Our electrode montage configurations and electrical field modeling predicted that either DC or low-frequency waveform at 1 mA will induce cortical and sub-cortical peak EF magnitude of ∼0.5 V/m. These values are similar to the ones generated in tES that has been shown to not only modulate cortical excitability and related physiological function (Thibaut et al., 2017; Guerra et al., 2018) but to also have therapeutic effects (Lefaucheur et al., 2017). Additionally, we note that our results hold if higher intensity pulses are used, as the quasi-static field approximation implies linearity of the induced EF magnitude solution. This implies, for instance, that 10-mA stimulation will induce 10 times the EF magnitude induced at 1 mA.

Beginning with the cortical surface EF plots that help to provide a general overall current flow picture (Figures 2, 4) followed by a quantitative comparison using percentage OE volume plots above EF threshold (Figure 3), we clearly demonstrate the benefit of montages involving nose bridge electrodes to better deliver current to our ROI. The consideration of additional drivers of polarization helped demonstrate that each optimal configuration results in a similar induced voltage profile in the OE but with differences in magnitude (Figure 5). As a result, the location of the axon plays a more important role than the actual montage considered in this sub-set of configurations. Further, we generally noted peaks toward the ends of axon, and, as expected, EF along axon peaks occurred in approximately the same axon segment as the activating function peaks. The discontinuities noted in the plots are likely driven by the overall current flow and not due to any change in electrical conductivity and tissue, as each exemplary axon was wholly contained within the OE mask. It is beyond the scope of this study to judge whether these values would induce action potentials as it would depend on the knowledge of activating function thresholds. These threshold values depend on the biophysics of the fibers (diameters, membrane resistance, etc.) and, moreover, different fibers will have different thresholds. We also note that due to the finite length of the axons in the OE, we can expect EF along the axon to be more relevant than the activating function.

The consideration of cross-sectional plots revealed that the optimal montages that are likely to modulate OB and OE also have the potential to influence the hippocampal and parahippocampal regions including EC (Figure 6). Montages 1, 4, and 6 were all capable of inducing relevant EF magnitude for modulating OB and 1/3 anterior sub-segment of OE. In the context of hair-free configuration of electrodes of Montage 6 and its superiority of effecting posterior 1/3 sub-segment of the OE in comparison to the other hair-free Montage 4, Montage 6 was more efficient to stimulate primary structures of the sensory system of the olfaction.

The stimulation of the EC as the end terminal of the OB neurons and the reported region of early pathogenesis in AD is a bottleneck for non-invasive neurostimulation modalities because of its anatomically deep localization in the cranium. It is worth to note that the stimulation of OB and OE can also be capable of stimulating the LEC selectively since the LEC is the secondary center for the OE and OB’s neural connections. In this context, Montages 1, 4, and 6 are all capable of stimulating the LEC selectively. In addition to this neural connectivity-based stimulation of the LEC via the OB and OE, the direct effect of the electrical current fields on the hippocampal and parahippocampal/EC regions including the LEC are also demonstrated in the Montages of 1, 4, and 6. However, Montages 4 and 6 demonstrated more prominent and more selective current flows over the hippocampal/parahippocampal/EC regions in comparison to Montage 1.

The OFC is known as the multisensory, cross-modal interaction center for olfaction and gathers olfactory information from the EC. All three montages were capable of directing the current to the OFC, and we did not observe any major differences. In the context of primary (OB, OE), secondary (EC), and tertiary (OFC) olfactory system structures, Montage 6 showed the most specific effect for the olfactory system and the least influence on the other structures in comparison to other montages. While Montage 1 was more efficient on the 1/3 middle and 1/3 posterior OE than Montage 6, there were no observable differences of current flow on the 1/3 anterior OE and on the entire OB as well as the OFC in between these two montages. However, Montage 1 had lesser impact on the hippocampal/parahippocampal/EC regions than Montage 6. In addition, it had a widespread effect on other cortical structures including motor/sensory cortices as well as a prominent influence on the occipital cortex. In this context, Montage 1 was not specific as Montage 6 for the early AD pathology-related anatomical regions and olfactory system structures.

Due to the dominant downward trajectory of current flow for Montages 4 and 6, higher EF was induced in the hippocampal and parahippocampal/EC and terminated at the cerebellum. However, Montage 1 directed the current to the overall cerebrum with relatively less impact on the hippocampal/parahippocampus/EC and more impact on the motor/sensory cortex (in comparison to Montages 4 and 6) and the current terminating at the occipital cortex but not in the cerebellum (Figures 4, 6). In the context of Montage 6’s capability of directing the current over the OB, 1/3 anterior and 1/3 posterior sub-segments of the OE, hippocampal/parahippocampal/EC, and OFC through the hair-free electrode placements, we can clearly postulate Montage 6 as the most ideal configuration for a spectacle like wearable stimulators to be tested on early stages of AD that demonstrated olfactory dysfunction. The current flow pattern due to Montage 6 has the potential to restore the sense of smell in AD; however, a more significant effect of this type of stimulation on AD can be observed because of the stimulation of the end terminals of olfactory nerve (like EC) that have cardinal roles in the pathophysiology of AD. As indicated previously, it has been shown that EC is primarily affected in preclinical AD (Khan et al., 2014) and tightly related to memory deficiencies in AD (Nagy et al., 1996). In this context, it can be postulated that EC stimulation via olfactory stimulation may improve EC function, and this can reflect on the memory and navigation functions in AD.

It is inevitable that any cutaneous/scalp electrical stimulation modality will likely stimulate non-specific structures like sensory and motor nerves of the skin, facial/scalp muscles, etc. As a result, the non-invasive electrode placements proposed in this study may activate additional non-target structures. This is expected given that injected current has to traverse through more tissue layers (skin, skull, etc.) than some of the approaches used thus far for targeting olfactory regions (transethmoidally, internasally, etc.). Furthermore, the proximity of the optimal montages to the eye regions increases the likelihood of visual phosphenes. While the safety and tolerability of any novel approach have to be carefully evaluated, it is to be noted that certain transcranial approaches, given the application, have actually exploited these high-conductivity pathways to intentionally target these sensitive regions (Haberbosch et al., 2019). Fortunately, not only stimulation parameters (current density, charge density, etc.) but also computational models approximating these conditions can be employed in combination to check against established safety limits. Only after confirmation of not exceeding these aforementioned limits should any clinical testing be attempted.

Computational studies like these provide a rational option to determine/screen optimal electrode placements in a prospective fashion. In a recent paper, Iravani et al. (2020) demonstrated that OB activity can be recorded by EEG electrodes that are placed on the forehead. Although the simulation method and approach of the present study is validated in the previous studies including human intracranial recordings (Huang et al., 2017), the OB responses to the montages that are presented in the present study have to be investigated in live human studies in combination with EEG and its potential beneficial effects on the neurological diseases and conditions including AD, PD, and vegetative states.

## Data Availability Statement

The original contributions presented in the study are included in the article/supplementary materials, further inquiries can be directed to the corresponding author/s.

## Author Contributions

YC and AD developed the concept idea. KN, CT, AD, and YC performed the literature review, determined the electrode configurations for the six montages to be simulated, analyzed the results, wrote and edited the main manuscript, post processed the results, and generated the diagrams. KN, CT, and AD ran the electrical field modeling for the six montages. All authors contributed to the article and approved the submitted version.

## Conflict of Interest

YC is a shareholder in Stoparkinson LLC. AD, CT, and KN are employees of Soterix Medical, Inc.
